# Tumorigenesis, diagnosis, and therapeutic potential of exosomes in liver cancer

**DOI:** 10.1186/s13045-019-0806-6

**Published:** 2019-12-09

**Authors:** Hongbo Wang, Zaiming Lu, Xiangxuan Zhao

**Affiliations:** Department of Radiology, Shengjing Hospital of China Medical University, 36 Sanhao Street, Heping District, Shenyang, 110004 Liaoning China

**Keywords:** Hepatocellular carcinoma, Exosomes, Tumor markers, Targeted therapy, Signal transduction

## Abstract

Hepatocellular carcinoma (HCC, also called primary liver cancer) is one of the most fatal cancers in the world. Due to the insidiousness of the onset of HCC and the lack of effective treatment methods, the prognosis of HCC is extremely poor, and the 5-year average survival rate is less than 10%. Exosomes are nano-sized microvesicle and contain various components such as nucleic acids, proteins, and lipids. Exosomes are important carriers for signal transmission or transportation of material from cell to cell or between cells and tissues. In recent years, exosomes have been considered as potential therapeutic targets of HCC. A large number of reports indicate that exosomes play a key role in the establishment of an HCC microenvironment, as well as the development, progression, invasion, metastasis, and even the diagnosis, treatment, and prognosis of HCC. However, the exact molecular mechanisms and roles of exosomes in these processes remain unclear. We believe that elucidation of the regulatory mechanism of HCC-related exosomes and its signaling pathway and analysis of its clinical applications in the diagnosis and treatment of HCC can provide useful clues for future treatment regimens for HCC. This article discusses and summarizes the research progress of HCC-related exosomes and their potential clinical applications.

## Background

Hepatocellular carcinoma (HCC, also called primary liver cancer) is one of the most common cancers in the world. There are about 840,000 new cases of HCC and at least 780,000 people die of HCC every year [1]. Surgical resection, liver transplantation, local administration of radiation or chemical drugs, and combined therapy are the main HCC treatment schemes [2]. However, due to the insidiousness of HCC onset and the lack of specific early-stage markers, most patients are often diagnosed at the advanced stages of HCC, which is generally not suitable for surgery, and their survival time is generally only 6 months [3]. Radiofrequency ablation (RFA) is currently the first-line treatment for small HCC. It has similar efficacy as surgical resection and has the characteristics of minimal damage and rapid recovery. However, due to the uneven distribution of ablation energy in the tumor, killing of the distal or terminal cancer cells is not efficient, which in turn may cause residual tumors [4]. Transcatheter arterial chemoembolization (TACE) is another first-line treatment for advanced HCC; however, due to compensatory vascular proliferation in the tumor after hypoxia, the treatment efficacy is also unsatisfactory [5]. Sorafenib is the mainstream targeted treatment drug for advanced HCC; it can significantly prolong the median survival of patients, while drug resistance gradually develops in the later stage of treatment [6]. Therefore, identification of early stage-specific diagnostic markers of HCC and solving the problems of drug resistance and disease recurrence are urgent issues in clinical practice. The development and progression of HCC is an extremely complicated pathological process, and the underlying molecular mechanisms remain unclear. A large number of basic and clinical studies have shown that viral infection, alcoholic, and non-alcoholic liver toxicity are major causes of HCC, whereas its pathogenesis is unknown [2, 7]. In recent years, both in vivo and in vitro experiments have demonstrated that exosomes may play a key role in the onset, development, diagnosis, and treatment of HCC [8]. The cell types that produce exosomes in the liver are mainly hepatic parenchymal cells (such as hepatocytes), non-parenchymal intrahepatic immune cells [such as macrophages, dendritic cells, T/B cells, and natural killer (NK) cells], and various non-parenchymal liver stromal cells (such as stellate cells) [9]. Exosomes are employed by various viruses, including hepatitis B virus (HBV) and hepatitis C virus (HCV), to transmit viral RNA complexes to adjacent normal liver cells, by plasmacytoid dendritic cells (PDC) to induce non-specific immune responses, and by T cells to inhibit specific immune responses [10–12]. The exosomes produced by hepatic stellate cells contain miR-335-5p, which can induce proliferation and inhibit invasion by reducing rho-associated protein kinase 1 (ROCK1) expression in target HCC cells [13]. However, it has also been reported that miR-21 carried in exosomes produced by HCC cells can transform hepatic stellate cells into cancer-associated fibroblasts (CAFs) to promote cancer progression [14]. Recent studies have also found that exosomal circRNAs produced by adipocytes of HCC patients can promote the growth and survival of HCC cells [15]. HCC cells can also evade tumor immune surveillance by generating exosomes for immune response inhibition [16]. Clinical studies have found that circulating exosomal non-coding RNAs (such as miR-21 [17], miR-122 [18], and lncRNA-ATB [19]) or proteins [such as galectin-3-binding protein (G3BP) [20], HMGB1 [16], LOXL4 [21], and HSP [22]] in the serum of HCC patients can be used as independent tumor markers for HCC staging and assessment of efficacy and prognosis. Therefore, exosomes play a key role in the development, diagnosis, and treatment of HCC and have a wide range of clinical applications.

## Overview of exosomes

Almost all human cells produce a type of lipid membrane-coated vesicle that can be secreted outside the cells, which is called an extracellular vesicle (EV) [23]. Based on its biogenesis and diameter, EVs fall into three main categories: microvesicles/ectosomes (100–1000 nm), exosomes (30–150 nm), and apoptosomes/apoptotic bodies (100–5000 nm) [24]. Among these, exosomes originating from intracellular endosomes are a type of heterogeneous intraluminal vesicles (ILVs) secreted outside the cell membrane [25]. The production, secretion, transport, uptake, and release of exosomes are regulated by specific signaling pathways and have strict spatial and temporal characteristics, which are an active and energy-consuming mechanism for maintaining intracellular homeostasis and involved in various physiological and pathological key life processes [26, 27].

### Features of exosomes

Exosomes are extracellular tiny vesicles with a lipid bilayer membrane structure, first discovered by Johnstone in 1989 while studying reticulocytes [28]. Exosomes are currently considered to be disc-shaped or cup-shaped extracellular vesicles with a diameter of about 30–150 nm and a density between 1.10 and 1.21 g/mL. Exosomes are nanoscale particles with various biological functions and widely distributed in body fluids such as blood [29], saliva [30], milk [31], ascites [32], and urine [33], which are rich in nucleic acids, proteins, lipids, and inorganic salt ions. Exosomes are involved in the exchange of important information and substances between cells or between cells and tissues. They maintain the key physiological processes of development, growth, differentiation, and aging of human cells and are necessary for human life. A growing number of laboratory and clinical studies have shown that secretion and function abnormalities of exosomes play a vital role in the development, progression, and treatment of malignant tumors [34].

### Formation and secretion of exosomes

**Fig. 1:**
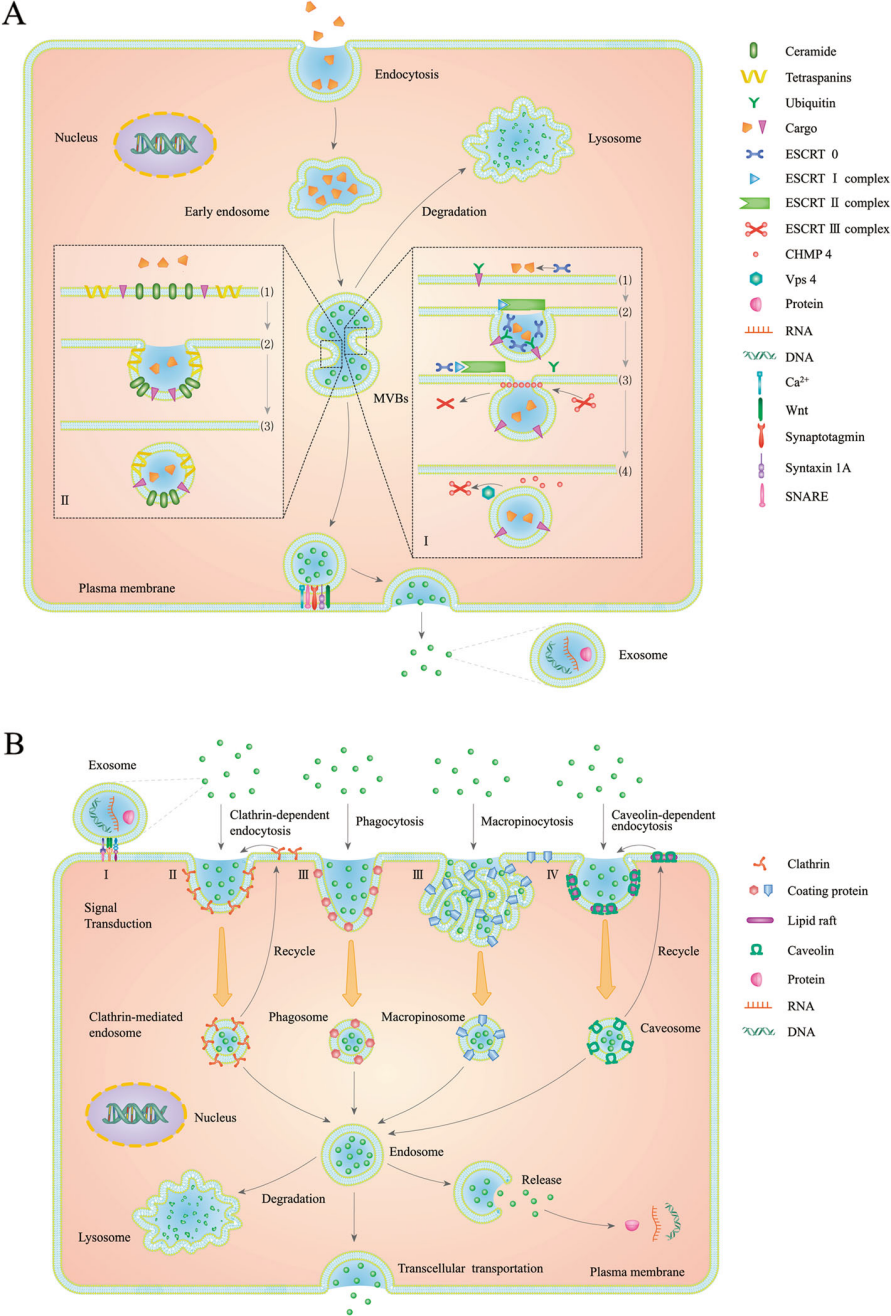
Schematic diagram of the main production and uptake mechanism of exosomes. **a**. The formation of exosomes. Endocytosis encapsulates the substances to form an early endosome that continue to mature into MVBs containing ILVs. (I) ESCRT-dependent pathway: (1) Ubiquitinated cargo activates nearby ECSRT0 to recruit more cargos to the vicinity. (2) Activated ESCRT0 further activates downstream ESCRTI and ESCRTII to form a large polymeric complex that further closes the lipid membrane invagination. (3) ESCRTII then induces the activation and polymerization CHMP4 subunit of ESCRTIII, which can lead to the formation of ILV. (4) The formation of ILV activates the nearby Vps-4 complex to dissociate CHMP4 from the lipid membrane into recycling. (II) ESCRT-independent pathway: (1) Cargo located close to the endosome lipid membrane enters the lipid membrane microdomains enriched in ceramide. (2) Cargo is enveloped in the invaginated pocket with the assistance of various tetraspanins, transmembrane proteins and lipids and (3) eventually forms ILV. MVB containing ILVs is transported to the vicinity of lysosome for degradation. MVB can also be delivered to the vicinity of specific plasma membrane, and anchor and fuse to the plasma membrane to secret the ILVs to form exosomes. **b**. Association and uptake of exosomes. (I) Exosomes can recognize and bind to the recipient cells and transmit specific signals. (II) Exosomes enter target cells by clathrin-dependent endocytosis. (III) Exosomes enter target cells by clathrin-independent macropinocytosis or phagocytosis pathway. (IV) Exosomes enter cells by lipid rafts, such as caveolae, mediated endocytosis. Depending on the needs of the cells, the exosomal contents are released into the cytosol, the exosomes are transported to the lysosome for degradation and digestion, or the exosomes fuse with cell membrane again and are released to accomplish the transcellular transport

Exosomes are initially formed by engulfment of the cell membrane to form early endosomes. These early endosomes can collect molecular materials such as target proteins, RNA, and DNA along the movement pathway as needed and further process, modify, and sort these materials. Endosomes then mature into multivesicular bodies (MVBs, also known as late endosomes) containing ILVs. Depending on the needs of the cells, MVBs can fuse with the cell membrane to release the ILVs to the extracellular space to form exosomes or with lysosomes to digest and degrade contained materials for material recycling (Fig. 1a). The identified formation processes of exosomes are mainly the endosomal sorting complex required for transport (ESCRT) pathway and the ESCRT-independent pathway [35, 36] (Fig. 1a). The ESCRT complex superfamily that is composed of more than 20 proteins consists of mainly five types of proteins: ESCRT-0/I/II/III and vacuolar protein sorting associated protein 4 (Vps4) [37–40]. In the ESCRT-dependent pathway, the ESCRT protein family plays an important role in the formation of exosomal ILV. First, the cargo in the membrane microdomain, which is rich in phosphatidylinositol 3-phosphate (PI3P), is ubiquitinated. The vicinal ESCRT-0 binds to ubiquitinated cargo through its zinc finger domain (ZFD) and ubiquitin-interacting motif (UIM) to initiate budding of the endosome lipid membrane. Studies also have found that the ESCRT-0 protein is a heterodimeric or tetrameric complex consisting two protein subunits, hepatocyte growth factor-regulated tyrosine kinase substrate (HRS) and signal-transducing adaptor molecule (STAM). HRS and STAM both contain two UIM functional motifs, and therefore it is possible for ESCRT-0 to simultaneously bind to eight ubiquitinated cargos. The HRS in the ESCRT-0 complex can further recruit ESCRT-I and ESCRT-II. ESCRT-I is a hetero-tetrameric complex composed of four protein subunits, namely tumor suppressing gene 101 (TSG101), vacuolar protein sorting-associated proteins Vps28, Vps37, and multivesicular body 12 (MVB12). Next, ESCRT-I combines with ESCRT-II and activates ESCRT-II. Activated ESCRT-II directly binds to the ESCRT-III protein subunit, charged multivesicular body protein 6 (CHMP6), and recruits another protein subunit, CHMP4 (also known as Vps32), to induce the assembly of ESCRT-III on the membrane of endosomes. CHMP4 forms a ring-like polymer complex around the engulfed neck of the budding, allowing the budding pocket to be tightened and closed. As CHMP3 is further added to the complex, budding is completely disconnected from the endosome lipid membrane and is dragged into the endosome lumen to form an ILV [35, 41, 42]. After the completion of lipid membrane remodeling under the action of ESCRT-III, additional Vps4 (a complex mainly composed of SKD1, LIP5, and CHMP5) is required to detach the ESCRT-III complex from the lipid membrane and disassemble each subunit to enter the next round of ILV formation (Fig. 1 a). The ESCRT-independent pathway was discovered by Trajkovic et al. when they blocked the ESCRT pathway using specific drug and found that the proteolipid protein (PLP) transport in exosomes was not affected [43]. Stuffers et al. further confirmed the existence of the ESCRT-independent pathway by deleting the expression of four types of ESCRT complexes and found that exosome-mediated CD63 secretion still could not be blocked [44]. However, at present, the detailed mechanism of the ESCRT non-dependent pathway remains unclear. Some studies have indicated that the ESCRT-independent pathway is regulated by ceramide. One of the possible mechanisms is that the neutral type II sphingomyelinase (NSMase II) can hydrolyze sphingomyelin to form ceramide, and the resulting ceramide aggregates to further form a membrane micirodomain depression [45], and ceramide can also be converted to sphingosine 1-phosphate to activate the G_i_-protein-coupled sphingosine 1-phosphate receptor to complete the carto sorting into the ILVs [46]. Therefore, the regulation of NSMase activity can control the formation of exosomes in the non-ESCRT pathway. For example, the compound GW4869 is a NSMase inhibitor, which specifically increases the secretion of EVs larger than 100 nm in diameter and reduces the secretion of small EVs (< 100 nm) by inhibiting SMPD2/3 (sphingomyelin phosphodiesterase 2/3) [47]. The molecular mechanism and the biological significance of exosome synthesis regulation and the increase in the number of large-particle EVs as mediated by NSMase inhibition in HCC remain unclear. In addition to ceramide, a family of small molecule 4-transmembrane proteins (tetraspanins) are also involved in the formation of ESCRT-independent exosomes, of which CD9 [48], CD63 [49], CD81 [50], CD82 [51], and CD97 [52] have been clearly identified. Mechanism analysis shows that these small molecule tetraspanins can interact with other transmembrane proteins, cytosolic proteins, or cholesterol to aggregate and form invaginated pockets [53, 54], as well as interact with various cargos (such as integrins) that are about to enter the exosomal pathway to direct them into ILVs and determine their destinies (formation of exosomes or degradation by lysosomes) [55, 56]. The current detailed molecular mechanism underlying the involvement of tetraspanins in the formation of exosomal ILVs remains elusive. Second, MVBs carrying ILVs are directed to the cell membrane, anchor in, and fuse with the cell membrane, releasing internal ILVs to the outside of the cell to form exosomes. Cytoskeleton proteins (such as actin and microtubules) [57], molecular motors (such as dynein, kinesin, and myosin) [58], and molecular switches (such as small GTPase [59]) participate in the transport of MVBs in cells [60]. One view believes that MVBs involved in exosome secretion generally move along microtubules from the negative end to the positive end; conversely, if MVBs move from the positive end of the microtubule to the negative end, then they will fuse with lysosomes [61]. Moreover, protein factors such as Rab GTPase Rab7 [62], Rab27a/b [63], Rab11 [64], Rab35 [65], synaptotagmin-like protein 4 [66], and exophilin 5 [67] are also involved in the directional movement and anchoring of MVBs in the cell membrane. Another view is that the lipid and protein composition of the lipid membrane microdomain at the time of ILV formation determines the destiny of MVBs. For example, cholesterol in the endosome lipid membrane microdomain recruits ubiquitinated Rab7, which determines whether MVBs are transported to lysosomes, whereas MVBs involved in exosome secretion are usually rich in cholesterol and un-ubiquitinated Rab7 in its internal ILV lipid membrane [68, 69]. Eventually, MVBs move to the cell membrane to fuse with it to release ILVs that form exosomes, and this process is regulated by the SNARE protein complex [70], syntaxin 1A [71], synaptotagmin protein family [72], Wnt [72], and Ca^2+^ [73]. The Rab GTPase family of proteins are important regulators of intercellular ILV transport, which affect vesicle transport by interacting with the cytoskeleton [60, 74]. Knocking out Rab27 results in the accumulation of intracellular vesicles near the nucleus, which then affects the secretion of intracellular vesicles [75]. It is currently believed that Rab GTPase-mediated regulation of exosomes may participate in the disposal of cellular materials or intercellular signaling [76–78]. However, the detailed molecular mechanism of the secretion of exosomes as regulated by Rab GTPases is not clear.

### Capture and uptake of exosome

When exosomes reach their target cells, they are mutually recognized and associate with membrane proteins such as tetraspanins [79], integrins [80], lectins [81], and heparan sulfate proteoglycans (HSPGs) [82], lipid molecules [83], and extracellular matrix components [80]. Such interaction may result in any of two outcomes. One is to stay on the surface of the cell membrane and activate the corresponding signaling pathways. For example, exosomes derived from B lymphocytes and dendritic cells can bind to T lymphocytes and present antigens to T cells, thereby inducing specific antigen responses [84]. Another outcome is to be taken up by the target cells [85] (Fig. 1 b). Endocytosis is currently considered one of the important uptake mechanisms of exosomes and can be divided into various forms, namely clathrin-dependent [86], clathrin-independent (mainly referring to macropinocytosis [87] and phagocytosis [88]), and caveolae or lipid raft-mediated [89] pathways (Fig. 1b). The receptor cell types and the components harbored by the exosomes determine the destinations of the exosomes. In the clathrin-dependent pathway, after the association of exosomes to the surface of the target cells, a grid-like clathrin protein complex composed of six protein molecules accumulates underneath the membrane, and the cell lipid membrane begins to invaginate to form a clathrin-coated pocket. GTP-binding dynamin aggregates at the neck of the pocket to form an annular band. As GTP hydrolyzes, the annular band further contracts to completely cleave the pocket from the cell membrane to form a clathrin-mediated endosome. The clathrin protein then dissociates from the coated vesicle, allowing it to fuse with the early endosome in the cell for subsequent sorting. Phagocytosis of the second clathrin-independent pathway is also a type of signal-mediated internalization. When the ligand recognizes the receptor, the local cell membrane invaginates through the action of microfilaments/actin filaments and its related proteins to wrap and internalize the cargo to form a phagosome. Phagocytosis can be accompanied by or without extension of the membrane, and the ingested exosomes are usually cell-specific [90]. The downregulation of actin expression and inhibition of PI3K activity can significantly block phagocytosis of exosomes [87]. Macropinocytosis is a cellular process involving exosome uptake that forms a macropinosome through the invagination of the plasma membrane to form folds. Macropinocytosis is similar to phagocytosis, whereas the extension of the cell membrane is necessary during the process of micropinocytosis, and mutual recognition with the internalized products is not required. During the formation of membrane folds, cholesterol on the cell membrane promotes the aggregation of Ras-related C3 botulinum toxin substrate 1 (Rac1) at the site of macropinocytosis, and therefore, Rac1 and cholesterol play important roles in the macropinocytosis of exosomes [91, 92]. The third special lipid raft structure caveolae-mediated endocytosis is also a mechanism of exosome uptake that has been extensively studied. Lipid rafts are dynamic membrane microdomains that are rich in cholesterol and sphingomyelin, with a diameter of about 70 nm and contain various signaling molecules and protein receptors. When lipid rafts bind to scaffolding proteins CAV1 (caveolin-1), CAV2 (caveolin-2), or CAV3 (caveolin-3), these form cell membrane invaginations called caveolae. After the exosomes enter the caveolae, annular bands are also formed by dynamin contraction to detach the caveolae from the plasma membrane. The caveolae bind to intracellular caveosomes to form early endosomes. The caveosomes can also be directly transferred to the other side of the cell, during which the caveolin scaffolding protein does not dissociate from the caveolae structure containing exosomes [92, 93]. Exosome uptake via endocytosis is an energy-consuming process [94]. Moreover, membrane proteins and the cytoskeleton are extremely important in the uptake of exosomes. After treating cells with proteinase K and cytochalasin D, exosome uptake is significantly reduced. However, these treatments do not completely block the uptake of exosomes [95, 96], thereby suggesting that other mechanisms may be involved in this mechanism. Exosomes entering the recipient cells either form MVBs that bind to lysosomes and are degraded or release their contents into the cytosol. Currently, the molecular mechanisms by which exosomes release their contents remain unclear.

## Exosomes and precancerous liver diseases

Because exosomes can mediate intercellular signal transduction and regulate a variety of pathological biological behaviors, recent studies have focused on the functions and molecular mechanisms of exosomes in the development and progression of cancers. Previous reports have shown that exosomes are involved in the development and progression of various precancerous liver diseases, including viral hepatitis, alcoholic liver disease, and fatty liver disease and are even involved in the progression of liver fibrosis, and ultimately the development of HCC.

### Viral hepatitis

Viral hepatitis is the most common liver infectious disease worldwide. In Asia, hepatitis B virus (HBV) infections are one of the most common predisposing factors for HCC. In developed countries such as Europe, North America, and Japan, hepatitis C virus (HCV) infections are the most common liver infectious disease. Cirrhosis caused by hepatitis virus infections is considered as one of the most direct factors that contribute to the development of HCC [2]. After the human body is infected with hepatitis virus, the exosomes produced by the infected liver cells contain viral nucleic acids and proteins, and thereby the viruses can further transfect into normal liver cells through the exosomes, resulting in the spread of infection. Therefore, exosome mediates the spread of hepatitis virus [97, 98]. There is evidence that Rab27a, a member of the Rab GTPases protein family, participates in the regulation of hepatitis virus replication [99]. For example, infected cells can excrete miR-23b, which imparts a tumor suppressor effect via the Rab27a-mediated exosomal pathway to restrict the virus from further invasion and infection. Therefore, such exosomes may be a mechanism of the infected liver cells to restrict viral replication and maintain homeostasis [76]. Because Rab27a is an important regulator of intercellular ILVs transport, the secretion of exosomes as mediated by the Rab family may play an important role in the spread of hepatitis virus. HBx is an important transcriptional regulatory protein of HBV. It can increase the secretion of exosomes containing HBV nucleic acids and proteins by regulating the activity of NSMase II, which in turn induces hyperproliferation of hepatic satellite cells (HSCs) [100, 101]. Therefore, HBx can promote the spread of HBV and then induce cell mutations, demonstrating a strong carcinogenic activity. The exosomes produced by HCV-infected hepatocytes contain large amounts of miR-19 and miR-192, which have also been shown to induce excessive proliferation and differentiation of HSCs, ultimately leading to liver fibrosis [102, 103].

After the hepatocytes are infected by the hepatitis viruses, the exosomes these produce mediate the spread of the hepatitis viruses, as well as activate the body’s immune functions to cope with the infection [12, 104]. In one respect, HBV infection can stimulate hepatocytes to produce exosomes containing the HBV genome. These exosomes can be captured by human immune system effector cells, namely the NK cells, thereby activating its cell membrane PRR (pattern recognition receptors) and RIG-I, which inhibit the immune killing ability of NK cells and help HBV escape recognition by the host cells’ immune system. After HCV infection of hepatocytes, exosomes produced by hepatocytes can increase the number of regulatory T_R_ cells and reduce the proliferation of helper T_H_ cells, thereby weakening the viral immune responses of the body and preventing HCV clearance [105]. It has been reported that hepatocytes infected with HBV can produce exosomes containing miR-21, miR-192, miR-215, miR-221, and miR-222, which can inhibit T cells to secrete IL-21, an important inflammatory molecule of hepatitis immunity, which then further reduces the killing of HBV by the immune system [106]. The non-specific immune system of the human body, as the first initiated immune response after hepatitis virus infection, plays an indispensable role in hepatitis immunity. However, the HCV dsRNA in exosomes produced by hepatocytes can inhibit the innate immune response by inhibiting the activation of cellular TLR3 (Toll-like receptor 3) [107]. Therefore, after virus invasion, exosomes play an important signal transduction role in the interaction between hepatocytes and viruses and the immune system, which may become one of the first targets for future treatment schemes of viral hepatitis.

### Alcoholic liver disease

Exosomes are intimately related to pathological changes involving alcoholic liver disease (ALD). In vitro culture of primary hepatocytes and in vivo animal studies have confirmed that alcohol intake can significantly increase the secretion of exosomes by hepatocytes, and the increase in the expression of miR-192 in exosomes suggests the possibility of ALD [108]. The content of miR-122 and miR-155 in serum exosomes of patients with ALD are significantly increased, and the degree of elevation is linearly correlated with the extent of liver damage caused by alcohol. Such exosomes with high levels of expression of miRs can stimulate the proliferation of monocytes and promote the conversion of macrophages to M1 macrophages, which increases the secretion of inflammatory factors, ultimately leading to liver damage [109–111]. miR-19b and miR-92 in serum exosomes of ALD patients can further induce liver fibrosis after alcoholic liver damage [112]. Currently, the detailed pathological process of how exosomes regulate alcoholic liver disease is still unknown. The molecular mechanism of exosomes in the development, progression, and treatment of alcoholic liver disease requires further investigation.

### Non-alcoholic fatty liver disease

Changes in diet structure and lifestyle have been associated with an increase in the incidence of obesity and diabetes. In addition, the incidence of metabolic diseases in association with non-alcoholic fatty liver disease (NAFLD) continues to increase each year. NAFLD was originally considered as benign fat accumulation in the liver; however, recent studies have shown that it is a serious metabolic abnormality that can lead to liver damage and fibrosis [113, 114]. Exosomes may be potentially utilized as markers and key regulators in the development of NAFLD. For example, some studies have shown that lipotoxicity can result in a significant increase in the number of exosomes produced by hepatocytes, and these exosomes can promote the proliferation of M1 macrophages and stimulate the secretion of inflammatory factors, which in turn lead to the development of nonalcoholic steatohepatitis (NASH) [115]. The miR-122 in this type of exosomes is a potential non-invasive diagnostic marker for NASH [116], and miR-122 expression levels are positively correlated with the degree of liver fibrosis [117]. Hepatocytes after lipotoxicity can also produce exosomes containing miRNAs (such as miR-192), which promote the activation of hepatic satellite cells (HSCs) and induce liver fibrosis [118]. In addition, exosomes produced by intrahepatic fat cells can activate the TGF-β signaling pathway and upregulate the expression of fibrosis-related factors (TIMP-1 and integrin ανβ-5), which ultimately lead to the development of cirrhosis [119]. Therefore, the effects of exosomes in the development and progression of NAFLD may be a promising target for future treatment regimens of NAFLD.

### Hepatocirrhosis

Hepatocirrhosis (HC) is a diffuse liver damage with unknown pathogenesis and is one of the most common chronic progressive liver diseases. Exosomes initiate the development and promote the progression of HC. The activation of HSCs stimulated by exosomes is a critical step in HC pathologenesis. Exosomes secreted by activated HSCs contain large amounts of the CCN2 protein (also called connective tissue growth factor) and related mRNAs. Resting HSCs are activated after engulfing such exosomes, leading to enhanced proliferation and secretion of fibrotic factors, which induce the development of liver fibrosis. Therefore, CCN2 in serum exosomes is considered to play an important role in the pathological process of liver fibrosis and may function as a marker for assessing the risk of liver fibrosis to progress to HCC [120, 121]. Furthermore, a variety of integrins and HSPGs act as co-receptors of a single CCN2, co-regulating the transport of exosomes and the association of exosomes with HSCs [82, 122]. miR-199 or miR-214 can inhibit CCN2 expression by specifically binding to the 3′-UTR of the CCN2 mRNA. However, some studies suggest that the expression level of miR-199 and miR-214 are reduced in both liver fibrotic tissue and activated HSCs. Therefore, activating miR-199/214 can negatively regulate the fibrotic signaling pathway [123–125]. The above studies suggest that the inhibition of CCN2 expression may be one of the most promising methods for the treatment of liver fibrosis.

### Development of HCC and immunosuppression

The development of HCC is the result of long-term accumulation of gene mutations. Before turning into a tumor, these cancerous cells are already different from normal tissues or cells. Under normal circumstances, the immune system can recognize these differences and activate the immune response to clear the cancerous cells [2]. However, the actual situation is that tumor cells can deceive immune cells and escape immune surveillance, which is inseparable from the immunosuppression induced by tumor cells using various mechanisms, including exosomes [126, 127]. Some studies have shown that the 14-3-3 ζ protein in exosomes produced by HCCs can reduce the activation, proliferation, and differentiation of T cells, and induce more T cells to transform into regulatory T_R_ cells, which lead to T cell depletion. This pathway has been confirmed by several studies to be associated with tumor evasion of immune surveillance [128]. Cheng et al. found that exosomes produced by HCC cells could inhibit the activation of immune cells to lead to immune evasion by stimulating macrophages to increase the secretion of IL-6, IL-1β, IL-10, and TNF-α, activating STAT3 pathway, and increasing PD-L1 protein expression. Another study has found that melatonin treatment can reduce this immunosuppressive effect [129], suggesting that melatonin may be used as an immunotherapy adjuvant for HCC. How to restore the immunogenicity of HCC cells and re-establish the mechanism of immune clearance in vivo has become a major research direction for the complete cure of HCC.

## Exosomes and the metastasis and progression of HCC

**Fig. 2:**
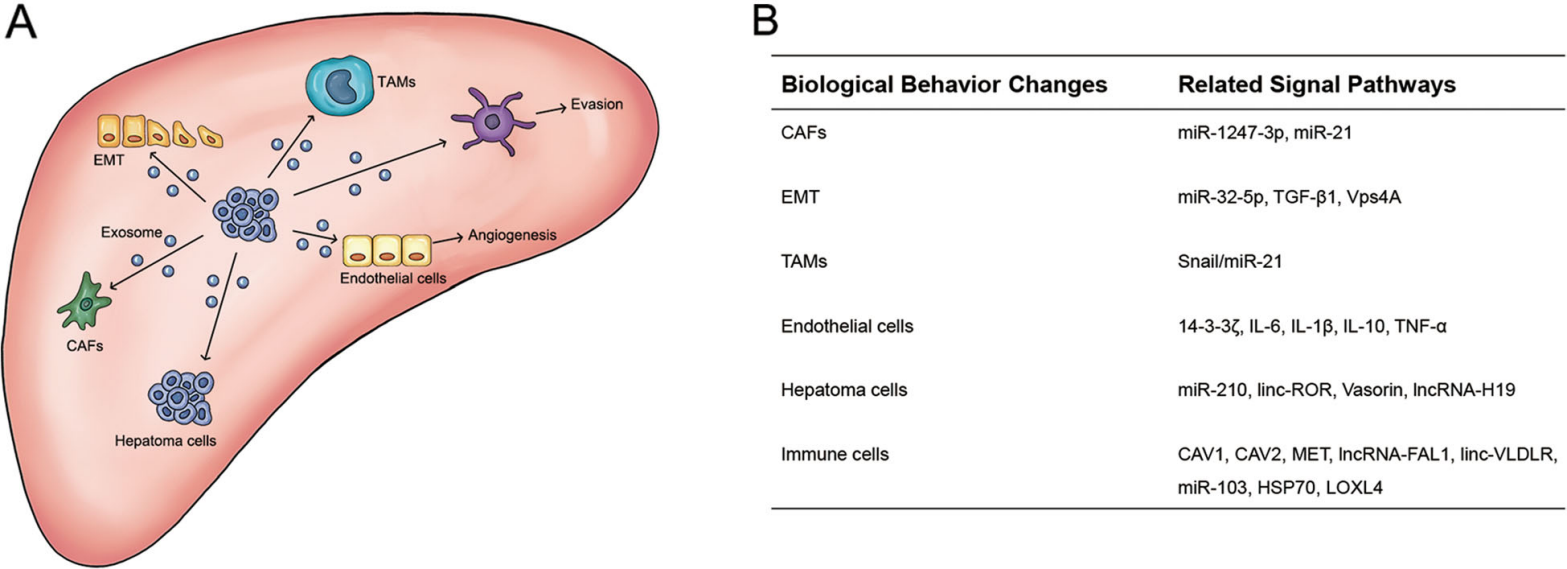
Hepatocellular carcinoma (HCC) cells can affect biological behavior changes of many types of cells by releasing exosomes. **a** Exosomes secreted by HCC cells can regulate EMT in adjacent microenvironment and the transformation of inflammatory microenvironment, coordinate with nearby tumor cells to increase invasiveness, and induce the conversion of adjacent fibroblasts and macrophages to CAFs and TAMs. Moreover, HCC-related exosomes can regulate the functions of immune cells and endothelial cells, to induce immune escape and angiogenesis. **b** HCC cell exosomes mediate signaling pathways and regulatory factors of intercellular interactions or interactions between cells and tissues

The signal molecules contained in the same type of exosomes that originate from various cancer cells are not the same, and the expression level of the molecules in the exosomes from one type of tumor cell is also different from those in serum and source cells [130]. Numerous experiments have confirmed that exosomes promote the metastasis and progression of HCC by regulating the tissue microenvironment and multiple signaling pathways in cancer and normal cells (Table 1 and Fig. 2).

**Table 1:** Cell biological behavioral changes and signaling pathways mediated by HCC-related exosomes

Cargo	Type	Source	Recipient Cell	Function	Mechanism	Ref
HMGB1	Protein	HepG2, Huh-7, Hep3B, LM3	B cell	Promote TIM-1(+) B cell expansion and suppress CD8(+) T cell activity	Activate TLR-MAPK pathway	[16]
LOXL4	Protein	SK-Hep1/LOXL4	SMMC-7721, SK-Hep1	Promote migration and angiogenesis	Activate FAK/Src pathway to alter cell-matrix adhesion and cell migration ability	[21]
Melatonin	Protein	HepG2, Bel7402	Macrophage	Reverse immunosupression	Suppress p-STAT3 and decrease PD-L1 expression	[129]
Vasorin	Protein	HepG2	HUVEC	Promote proliferation and migration	Not mention	[160]
GOLM1	Protein	MHCC97H	MHCC97H	Promote proliferation, migration and invasion	Activate GSK-3β/MMPs axis	[161]
SMAD3	Protein + RNA	Huh-7	Huh-7	Promote proliferation and adhesion	Enhance TGF-β-SMAD3-ROS signal	[147]
circ-DB	RNA	3T3L1	HepG2	Promote tumor growth and inhibit DNA damage	Suppress miR-34a expression and enhance USP7 and Cyclin A2 expression	[15]
circ-PTGR1	RNA	LM3	HepG2, 97L	Promote migration, invasion and metastasis	Activate MET via interacting with miR-449a	[202]
linc-ROR	RNA	HepG2	HepG2	Tolerance to hypoxia	Neutralize miR-145 and upregulate HIF-1α	[142]
linc-ROR	RNA	HepG2	HepG2	Induce chemoresistance	Not mention	[211]
linc-VLDLR	RNA	HepG2	HepG2, KMBC	Promote chemoresistance	Increase expression of ABC-G2 and ABC-C1	[212]
lncRNA-H19	RNA	Huh-7 (CD90+)	HUVEC	Promote tube formation and cell-cell adhesion	Increase VEGF and ICAM1	[146]
lncRNA-FAL1	RNA	HCC patients serum	Huh-7, HepG2	Promote proliferation and migration	Suppress miR-1236 and upregulate ZEB1 and AFP	[152]
miR-21	RNA	MHCC97H	LX2	Convert normal HSCs to cancer-associated fibroblasts	Depress PTEN, upregulate PDK1/AKT pathway and promote lipogenesis	[14]
miR-1247-3p	RNA	CSQT-2, LM3	Fibroblast	Promote tumorstemness, EMT, chemoresistance, tumorigenicity and metastasis	Downregulate B4GALT3 and activate β1-integrin/NF-κB axis	[134]
miR-125a/b	RNA	TAM	Huh-7, HepG2	Suppress proliferation, stem cell properties and migration	Decrease CD90 expression	[137]
miR-210	RNA	QGY-7703	HUVEC	Promote tubulogenesis	Inhibit SMAD4 and STAT6	[143]
miR-155	RNA	Huh-7, PLC/PRF/5	HUVEC	Promote tube formation under hypoxia condition	Not mention	[145]
miR-103	RNA	QGY-7703	HUVEC	Increase permeability of endothelial monolayers and facilitate transendothelial invasion	Decrease VE-Cad, p120 and ZO-1 to attenuate endothelial adhesion junction integrity	[148]
miR-32-5p	RNA	Bel7402/5-FU	Bel7402	Induce multidrug resistance via angiogenesis and EMT	Suppress PTEN and activate PI3K/Akt pathway	[149]
miR-25-5p	RNA	Huh-7, LM3	Huh-7, LM3	Stimulate transendothelial motility and enhance CTC tumor self-seeding ability	Inhibit LRRC7 expression	[150]
miR-93	RNA	HCC patients serum	SK-Hep1, Huh-7	Stimulate proliferation and invasion	Suppress CDKN1A, TP53INP1 and TIMP2	[151]
miR-122	RNA	AMSC	HepG2	Induce chemosensitivity and cell cycle arrest	Downregulate Cyclin G1, ADAM10 and IGF1R	[222]
miR-320a	RNA	CAF	MHCC97H, SMMC-7721	Inhibit proliferation and metastasis ability	Suppress PBX3/ERK1/2/CDK2 axis	[225]

*ABC* ATP-binding cassette, *AMSC* Adipose tissue-derived mesenchymal stem cell, *B4GALT3* β-1,4-galactosyltransferases III, *CAF* Cancer-associated fibroblast, *CDKN1A* Cyclin-dependent kinase inhibitor 1A, *circ* Circular RNA, *EMT* Epithelial to mesenchymal transition, *HCC* Hepatocellular carcinoma, *HUVEC* Human umbilical vein endothelial cell, *Linc* Long intergenic non-coding RNA, *LncRNA* Long non-coding RNA, *LRRC7* Leucine-rich repeat-containing protein 7, *miR* microRNA, *PBX3* Pre-B-Cell Leukemia Homeobox 3, *TAM* Tumor-associated macrophage, *TIMP2* Tissue Inhibitor of Metalloproteinase-2, *TP53INP1* Tumor protein p53-inducible nuclear protein 1, *ZEB1* Zinc finger E-box binding homeobox 1

First, exosomes participate in HCC microenvironment remodeling. Epithelial-mesenchymal transition (EMT) is a process in which cells gradually lose their epithelial morphological characteristics and transform into mesenchymal types, which is involved in tumor progression and metastasis [131]. Studies have found that exosomal miR-140-3p produced by HCC can inhibit MAPK/ERK pathway activity; increase the expression of actin α (α-SMA), vimentin, and N-cadherin; and reduce the expression of E-cadherin, ultimately inducing EMT and metastasis [132, 133]. The extracellular matrix (ECM) is a component of the tumor microenvironment, and ECM remodeling plays an important regulatory role in the development of HCC, similar to that of EMT. Cancer-associated fibroblasts (CAFs) and tumor-associated macrophages (TAMs), which are important ECM components, play an important role in the metastasis of HCC. The significantly increased expression of miR-1247-3p in HCC exosomes can lead to the downregulation of β-1,4-galactosyltransferases III (B4GALT3), activate the integrin β1/NF-κB pathway, and induce the transformation of fibroblasts to CAFs. These CAFs can secret inflammatory factors such as IL-6 and IL-8 to promote HCC progression [134]. It has been reported that miR-21 can induce the differentiation of monocytes into M2 TAMs by inhibiting the expression of programmed cell death protein 4 (PDCD4) and IL12A [135]. The expression of TGF-β1 in these TAMs is relatively high, which can further induce EMT, promote the proliferation of cancer stem cells (CSCs), and enhance the invasiveness of HCC cells [136]. Wang et al. suggested that the low expression level of miR-125a/b in TAM exosomes might be associated with the characteristics of CSCs [137], whose specific molecular mechanism awaits further experimental verification. Second, exosomes participate in HCC neovascularization. It is well known that due to the rapid proliferation of cancer cells, as the tumor volume increases and the blood supply becomes insufficient, internal cells are often in a hypoxic state. Stimulated by hypoxic conditions, tumor cells can activate the corresponding pathway via exosomes that promote neovascularization in response to hypoxic stress [138, 139]. Hypoxia-inducible factor-1α (HIF-1α) is an important regulator of cells in responses to hypoxic conditions, which regulates the function of endothelial cells via the VEGF/VEGFR pathway [140, 141]. Exosomes can regulate HIF-1α expression level by transporting linc-RoR to cope with hypoxic conditions [142]. Moreover, miR-210 in exosomes produced by HCC inhibits the expression of SMAD4 and STAT6 in human umbilical vein endothelial cell (HUVECs) [143], and exosomes secreted by hypoxia-stimulated HCC cells enhance the expression of VEGF/VEGFR in endothelial cells, both of which can promote angiogenesis [144]. Exosomal miR-155 produced by hypoxia-stimulated HCC can induce neovascularization in HUVECs, and the upregulation of serum exosomal miR-155 in HCC patients has been associated with earlier recurrence [145]. The level of lncRNA-H19 in exosomes produced by CD90^+^ HCC cells is significantly increased, which can upregulate the expression of VEGF and promote the formation of tube-like structures of HUVECs [146]. Zhou et al. found that miR-21 in HCC exosomes could activate HSCs and promote the transformation of HSCs to CAFs by regulating the PTEN/PDK1/Akt pathway, and these CAFs are closer to newborn blood vessels than other cells, suggesting that miR-21-activated CAFs may promote HCC progression by participating in angiogenesis [14]. The presence of primary tumor lesions is essential for lung metastasis of HCC and may transmit certain signals via exosomes [147]. For example, exosomes that originated from the primary lesion transport miR-103 to endothelial cells, which in turn reduces the expression of multiple endothelial cell adhesion factors (VE-Cad, p120, and ZO-1), enhances vascular permeability, and thereby facilitating the movement of tumor cells across endothelial cells [148]. Drug-resistant HCC cells produce exosomes containing miR-32-5p that activate the PI3K/Akt pathway in sensitive HCC cells, ultimately conferring drug resistance. Furthermore, exosomal miR-32-5p can also increase N-cadherin expression and reduce E-cadherin expression in target cells to induce EMT. miR-32-5p is overexpressed in the exosomes of HCC tissue, which inhibits PTEN expression and induces angiogenesis. Patients with miR-32-5p overexpression and low PTEN expression usually have poor prognosis [149]. Exosomal miR-25-5p enhances the movement of tumor cells trespassing the endothelial cells by inhibiting the expression of cell adhesion and migration-related protein leucine-rich repeat-containing protein 7 (LRRC7). These tumor cells entering the circulation via the vascular endothelia become circulating tumor cells (CTCs) [150]. Third, exosomes directly stimulate the proliferation of cancer cells. For example, miR-93 is highly expressed in HCC exosomes, which in turn regulates the expression of tumor protein p53-inducible nuclear protein 1 (TP53INP1), tissue inhibitor of metalloproteinase-2 (TIMP2), and cyclin-dependent kinase inhibitor 1A (CDKN1A) to induce HCC proliferation [151]. Long non-coding RNA (lncRNA) can regulate the expression of competing endogenous RNAs (ceRNA). Studies have confirmed that lncRNA-FAL1 is overexpressed in tissues of patients with HCC, and the level of lncRNA-FAL1 in serum exosomes is also significantly higher than that in healthy people. lncRNA-FAL1 can competitively associate with miR-1236 to upregulate the expression of alpha fetoprotein (AFP), zinc finger E-box binding homeobox 1 (ZEB1), vimentin, and E-cadherin to promote the proliferation and metastasis of HCC cells [152], suggesting that lncRNA-FAL1 can be used as a diagnostic marker for HCC progression.

Furthermore, HCC-related exosomes contain a large number of membrane proteins and soluble proteins in addition to nucleic acid substances. Although they are not as stable as the latter, they are also involved in the regulation of HCC metastasis and progression. For example, the heat shock protein (HSP) family participates in the regulation of VEGF secretion, VEGFR stability, endothelial cell migration, angiogenesis, cell proliferation, immune evasion, and other processes during HCC metastasis [153]. In particular, HSP often enhances tumor immunosuppression when present in cell membranes or other extracellular locations (such as exosomes) [154]. Xiao et al. found that MS-275 could induce a significant increase of HSP70 and MICA/B in exosomes produced by HCC cell line HepG2, and these exosomes could enhance the cytotoxicity of NK cells to HCC cells and then inhibit the progression of HCC [126]. Zhu et al. found that HSP70, CD63, and transglutaminase 2 (TGM2) coexisted in exosomes produced by the HCC cell line Huh7 [155]. Studies have also shown that TGM2 interacts with exosome secretion-related proteins TSG101 and ALIX to participate in the sorting of exosome cargo [156]. However, the role of TGM2 in HCC progression and metastasis remains unknown. Rao et al. reported that HSP70, AFP, and glypican 3 contained in exosomes produced by HCC cells could function as immunoregulatory proteins, and such exosomes could stimulate the proliferation and differentiation of DC cells, thereby inhibiting the growth of tumor cells in vivo and in vitro [157]. Yukawa et al. reported that exosomal HSP70 and NKG2D produced by HCC cell lines induced co-cultured endothelial cell HUVECs to form vascular lumens [158]. Studies have also shown that the expression of HSP90 in serum exosomes of cancer patients with highly metastatic CCA is significantly higher than that in patients with common CCA, suggesting that the level of HSP90 in serum exosomes is related to the malignant degree of the tumor, probably due to the fact that HSP90 contributes to tumor metastasis and invasion by activating related proteins in the extracellular microenvironment. Further studies have also found that the phosphorylation of HSP90, especially a decrease in the phosphorylation of HSP90B protein at the Ser255 and Ser581 amino acid sites, is inversely correlated to the metastatic ability of cholangiocarcinoma cells [159]. Therefore, exosomal HPS90 and its phosphorylation state may be used as markers for the progression of hepatobiliary tumors. The HDAC inhibitor MS-275 can enhance the specific or non-specific anti-tumor responses by upregulating tumor antigen proteins, human leukocyte antigen-I, human leukocyte antigen-II, co-stimulatory molecule B7, and immune adhesion molecules. Studies have shown that HCC-secreted exosomes transmit vasorin to HUVECs to promote their proliferation, which in turn enhances tube formation [160]. The expression level of lysyl oxidase-like 4 (LOXL4) is related to the tumor staging of HCC patients and plays an important regulatory role in tumor progression and metastasis. Especially after exosomal LOXL4 is taken up by target cells, LOXL4 decomposes lysine to produce hydrogen peroxide (ROS), which in turn activates the FAK/Src pathway and promotes HCC cell migration [21]. Golgi membrane protein 1 (Golm1/Golph2/GP73) exists as both membrane protein and soluble secreted protein. Hepatitis virus infection of hepatocytes can significantly upregulate Golm1 expression. Clinical studies have shown that the expression of Golm1 in HCC tissues is significantly higher than in adjacent normal liver tissue, and exosomal Golm1 can induce cell proliferation, migration, and invasion to promote HCC progression via GSK-3β/MMPs [161]. Compared with tumor-adjacent tissues, HCC tissues demonstrate more TIM-1^+^ B_reg_ cell infiltration, probably because HCC produced exosomal HMGB1 can activate and promote the proliferation of TIM-1^+^ B_reg_ cells via the Toll-like receptor 2/4 (TLR2/4)-MAPK pathway, and the TIM-1^+^ B_reg_ cells express immunosuppressive IL-10 to strongly inhibit CD8^+^ T cell activity, contributing to immune surveillance escape of HCC cells. Clinical studies have also shown that TIM-1^+^ B_reg_ cell infiltration usually indicates advanced cancer with easy recurrence and short survival [16]. HCV infection can induce the overexpression of transferrin receptor 2 (TfR2) in hepatocytes, leading to increased iron uptake and excessive deposition of iron in the liver, which eventually lead to cirrhosis [162, 163]. Overexpressed TfR2 in HCC cells can be transported to other cells in the form of exosomal TfR2 through invagination of the lipid raft structure caveolae to form endosomes. Exosomal TfR2 can activate the p38MAPK and ERK1/2 signaling pathway in HCV-infected hepatocytes to promote carcinogenesis, and liver iron overload caused by TfR2 eventually leads to a greater range of cirrhosis and promotes HCC progression [164]. Studies have shown that when exosomal CAV1, CAV2, and Met protein produced by high motility HCC cell lines are captured by surrounding normal liver cells, the MAPK/PI3K/Akt pathway in target cells is activated, resulting in immortalization and migration, and when captured by HCC cells, the invasiveness of HCC cells increases, indicating that CAV and Met play important roles in HCC metastasis and progression [165, 166]. Vps4A, an important regulator of exosome synthesis, acts as a tumor suppressor by regulating exosome cargo sorting and the uptake process [167]. For example, Vps4A can increase the amount of β-catenin entering exosomes, and further reduce its content in nuclei, thereby inhibiting its transcription regulatory function. The expression of Vps4A in HCC tissue samples is usually low, resulting in more β-catenin entering the nuclei and thereby promoting HCC progression and EMT [168]. Furthermore, exosomes produced by HCC can induce phenotypic changes in adjacent cells, causing them to present higher affinity to tumor cells, thereby facilitating the development of tumors. For example, HCC exosomes can activate the NF-κB pathway in adipocytes and increase the synthesis of inflammatory mediators such as IL-6, IL-8, and MCP-1, providing a convenient environment for tumor development [169]. Fu et al. reported that exosomal SMAD3 produced by HCC can be transported to distant or surrounding region to facilitate survival and proliferation of CTCs after distant colonization to promote HCC metastasis. Therefore, the presence of SMAD3 protein in serum exosomes usually indicates that the tumor has metastasized [147]. Li et al. found that exosomes containing CXC chemokine receptor-4 (CXCR4) produced by high-mobility HCC can stimulate and promote migration and invasion of low-metastatic HCC cells in distant or surrounding region, which may be due to the fact that exosomal CXCR4 can mediate the secretion of MMP-2/9 in the ECM [170].

## Exosomes and HCC diagnosis

Because HCC often has no specific manifestations in the early stage, patients often miss the optimal treatment period. Therefore, early or extremely early diagnosis is the most important prerequisite for successful HCC treatment. In clinical studies, tumor markers (such as α-fetoprotein, AFP), imaging, and histopathological biopsies are commonly used diagnostic methods for HCC. Because about 50% of HCC patients are AFP-negative, AFP has low sensitivity and specificity for HCC screening [171]. Imaging has high specificity; however, its sensitivity is relatively low in that it is unable to distinguish very small tumors [172]. The clinical applications of histopathological biopsies are also limited due to its invasive nature and high false-negative rate [2]. Exosomes from different tumor sources contain different levels of molecules, and those in exosomes are also different from those in the original cytoplasm, which can be several times higher/lower. This difference is more pronounced when compared with healthy people. Relative to the complicated environment in tissues and cells, the environment within exosomes is relatively simple and stable, and the exosomes can carry various proteases or other enzymes to the desired targets through blood, and thus can be used for the diagnosis of cancer progression and metastasis [8, 9, 34, 130, 173]. The potential of levels of molecules inside exosomes as a marker for liver malignant tumors has attracted the attention of tumor biologists and clinicians and may later become a non-invasive liquid biopsy marker for HCC [18, 19, 29, 174, 175]. This paper summarizes the reported potential markers of HCC-related exosomes (Table 2).

**Table 2:** Tumor molecular markers with potential clinical value in HCC-related exosomes

Factors as biomarker	Potential Mechanism	Exosome isolation methods	Ref
circ-0008043, circ-0003731, circ-0088030↑	Promote migration, invasion and metastasis	Exoquick (System Biosciences, USA)	[202]
LINC00161↑	Promote tumor migration and invasion	Total exosome isolation kit (Invitrogen, USA)	[196]
LINC-000635, ENSG00000258332.1↑	Positive related to portal vein tumor emboli, lymph node metastasis and worse OS	Total exosome isolation reagent (Thermo Fisher Scientific Co. Ltd, USA)	[199]
lncRNA-FAL1↑	Promote proliferation and migration	Exoquick TC (System Biosciences, USA)	[152]
lncRNA-HEIH↑	Promote tumor progression	GS0301 (Guangzhou Geneseed Biotech Co, China)	[198]
lncRNA-RP11-513I15.6, mRNA-RAB11A↑, miR-1262↓	Associate with poor prognosis	ExoRNeasy® RNA isolation kit (Qiagen, USA)	[201]
miR-21↑	Correlate with tumor stage	Total exosome isolation reagent (From serum) (Invitrogen, USA)	[17]
miR-21↑, lncRNA-ATB↑	Short progression time and OS	Exoquick (System Biosciences, USA)	[19]
miR-155↑	Promote tube formation under hypoxia condition	Exoquick (System Biosciences, USA)	[145]
miR-93↑	Stimulate proliferation and invasion	Total exosome isolation reagent (Thermo Fisher Scientific Co. Ltd, USA)	[151]
miR-638↓	Inhibit cancer cells proliferation	Total exosome isolation kit (Invitrogen, USA)	[174]
miR-9-3p↓	Suppress proliferation	Ultracentrifugation	[176]
miR-92b↑	Promote migration and affect NK cell cytotoxicity via downregulating CD69	Exoquick (System Biosciences, USA)	[181]
miR-718↓	Suppresses cell proliferation via targeting HOXB8	Ultracentrifugation	[182]
miR-125b↓	Short TTR and OS	Exoquick (System Biosciences, USA)	[194]
mRNA-hnRNPH1↑	Positive related to poor differentiation and worse OS	Total exosome isolation reagent (Thermo Fisher Scientific Co. Ltd, USA)	[175]
G3BP↑, PIGR↑	Promote tumor progression, transformation, invasion and proliferation	Ultracentrifugation	[206]

↑: Upregulation, ↓: Downregulation, *circ* Circular RNA, *G3BP* Galectin-3-binding protein, *HOXB8* Homeobox B8, *LINC* Long intergenic non-coding RNA, *LncRNA* Long non-coding RNA, *miR* microRNA, *mRNA* Messenger RNA, *OS* Overall survival, *PIGR* Polymeric immunoglobulin receptor, *TTR* Time to recurrence

First, because microRNAs (miRNAs/miRs) are extremely abundant in exosomes and have stable properties that are difficult to be degraded, these can be used as ideal markers for the prediction of the development of HCC. For example, clinical studies have found that serum exosomal miR-9-3p levels in HCC patients are significantly higher than in healthy people. miR-9-3p can induce proliferation inhibition and even apoptosis in HCC cells by downregulating HBGF-5 (heparin-binding growth factor-5) and ERK1/2 expression. Therefore, exosomal miR-9-3p may be used for the diagnosis of HCC and as a potential therapeutic target [176]. miR-21 can promote the proliferation and metastasis of HCC cells by inhibiting the expression of PTEN [177], PDCD4, RECK, and hSulf-1 (human sulfatase-1) [178] and even confer chemotherapy drug resistance to HCC cells [179]. Therefore, it is considered an oncomir [180]. The level of exosomal miR-21 in the blood of HCC patients is significantly higher than that in chronic hepatitis B (CHB) patients or healthy people. This difference is more obvious than the difference of miR-21 in whole blood. Moreover, the elevated level of serum exosomal miR-21 is often positively correlated with tumor stage, and then exosomal miR-21 can be used as a potential diagnostic marker for HCC [17]. Clinical studies have shown that the content of exosomal miR-93 in the HCC cell culture medium and patient serum are significantly higher and is positively correlated with tumor stage and tumor size of HCC patients, but negatively correlated with overall survival. Therefore, exosomal miR-93 can be used as an independent indicator for the prognosis of HCC. Mechanism analysis has showed that exosomal miR-93 could significantly inhibit the expression of CDKN1A, TP53INP1, and TIMP2 in HCC cells, and then promote the proliferation, invasion, and metastasis of HCC cells [151]. The content of exosomal miR-92b in serum of HCC patients is significantly higher than that of healthy people. The level of miR-92b decreases after liver transplantation. Conversely, if the exosomal miR-92b level continues to rise, then it often induces premature recurrence [181]. Studies have reported that serum exosomal miR-718 can promote HCC cell proliferation by inhibiting PTEN expression. However, it has also been reported that the expression of serum exosomal miR-718 in HCC patients after liver transplantation is negatively correlated with the recurrence rate of HCC. One possible reason is that the lncRNA SEMA3B-AS1 in normal liver tissue after transplantation (which is usually expressed at a low level in HCC tissue) inhibits the function of miR-718, and therefore, screening of lncRNA SEMA3B-AS1 after liver transplantation may be very important [182, 183]. The tumor suppressor miR-122 can inhibit the proliferation of HCC cells by blocking the activation of Akt3 [184], Wnt [185], and Bcl-ω [186] and is expressed at a low level in HCC tissues. For HCC patients with cirrhosis, the lower the ratio of serum exosomal miR-122 before and after TACE treatment (after/before-TACE), the worse the prognosis. Therefore, the decrease of exosomal miR-122 level may function as a diagnosis indicator of significant treatment efficacy [18]. The expression of miR-638 in in vitro cultured HCC cells or patient tissues is significantly lower than that in normal hepatocytes or tissues. High expression of miR-638 inhibits the proliferation, invasion, and angiogenesis of HCC cells to exert tumor suppression effects by inhibiting sex determining region Y-Box 2 (SOX2) or reducing intracellular oxidative stress status [187–189]. The low expression of miR-638 in serum exosomes of HCC patients before treatment often indicates a short overall survival (OS) [175], suggesting that miR-638 can be used as one of the prognostic indicators. miR-125b can inhibit HCC cell proliferation by downregulating sirtuin7 [190], hexokinase 2 [191], and SIRT6 [192] and disrupting neovascularization to reduce HCC metastasis by downregulating the expression of Angpt2 (angiopoietin 2) [193]. Compared with normal liver tissues and hepatocytes, miR-125b expression in HCC tissues and in vitro cultured HCC cells is significantly reduced. Clinical analysis of miR-125b in serum of HCC, CHB, and liver cirrhosis (LC) patients showed that the level of miR-125b in serum exosomes was significantly higher than free miR-125b in serum in the three patient groups, and serum exosomal miR-125b in CHB patients was significantly higher than that in HCC patients. HCC patients with low level of serum exosomal miR-125b are often associated with early recurrence and shorter OS [194], indicating that miR-125b with anticancer effect can be used for clinical HCC efficacy diagnosis. In addition, although studies have found that serum exosomal miR-145, miR-192, miR-194, miR-29a, miR-17-5p, and miR-106a in HCC patients are significantly higher than in healthy people [195], and the level of miR-18a, miR-221, miR-222, and miR-224 in serum exosomes of HCC patients are significantly higher than those in patients with CHB infection or LC, the levels of miR-101, miR-106b, miR-122, and miR-195 are lower than those in CHB patients [29]. However, these miR studies still lack strong clinical and basic research data to confirm their effects in HCC diagnosis. In summary, the above studies show that HCC exosomal miRs play an important role in diagnosis and prognosis analysis, and some miRs have demonstrated a strong prospect of clinical kit application. However, clinical trials have shown that the sensitivity and specificity of single miRs in predicting the development of HCC are not ideal. Therefore, comprehensive detection and evaluation using multiple factors (e.g., miR-122 and miR-148a combined with AFP) may be able to more accurately distinguish early HCC from cirrhosis.

Second, lncRNAs, which are predominant RNA species in cells, show differences in expression levels in HCC cells and exosomes and also have potential diagnostic applications. The expression level of LINC00161 in serum exosomes of HCC patients is significantly higher than that in normal control group [196]. lncRNA high expression in HCC (lncRNA-HEIH) is significantly elevated in HCC tissues. Mechanism analysis shows that lncRNA-HEIH conjugates with enhancer EZH2 to regulate the expression of its target genes to promote cell cycle progression, and eventually to promote HCC progression, resulting in a poor prognosis in lncRNA-HEIH high expression patients [197]. Zhang et al. reported that the expression level of lncRNA-HEIH in serum exosomes of HCC patients was significantly higher than that of CHC and LC patients [198]. Therefore, lncRNA-HEIH may be potentially used in the diagnosis of HCC. Xu et al. reported that the expression levels of ENSG00000258332.1 and LINC000635 in serum exosomes of HCC patients were significantly higher than those of healthy people and LC and CHB patients, and exosomal ENSG00000258332.1 and LINC000635 were positively correlated with HCC lymph node metastasis and TNM stage, but negatively correlated with OS, and further studies showed that combined detection of these two with AFP could significantly improve the detection sensitivity and accuracy of HCC [199]. The expression of lncRNA-activated by tumor growth factor-β (TGF-β) (lncRNA-ATB) in HCC tissues is significantly higher than in adjacent normal tissues, and is significantly positively correlated with metastasis, staging, and tumor volume, but negatively correlated with OS. By competitive association with miR-200, lncRNA-ATB can upregulate ZEB1 and ZEB2 to affect HCC metastasis and invasion, and promote post-metastasis proliferation via IL-11 and STAT3 [200]. Similarly, Lee et al. reported that lncRNA-ATB contained in serum exosomes of HCC patients was also positively correlated with tumor TNM stage and volume, and negatively correlated with OS [19]. Therefore, exosomal lncRNA-ATB may be used as independent marker for HCC diagnosis and prognosis.

Third, mRNAs act as RNAs encoding proteins in humans, and the expression of mRNAs is clearly correlated with cellular functions. hnRNPH1 is a RNA-binding protein and a splicing factor that can alternatively splice mRNA, and its abnormal high expression plays an important role in the carcinogenesis and differentiation of hepatocytes [52]. Studies have shown that mRNA-hnRNPH1 level in exosomes of HCC patients is significantly higher than that in healthy people and LC and CHB patients. Its expression level is positively correlated with HCC stage and lymph node metastasis and negatively correlated with OS, and therefore, it has certain clinical diagnostic value. However, although hnRNPH1 is highly sensitive, considering the poor stability of it, co-detection of hnRNPH1 and AFP may make up for this disadvantage [175]. Furthermore, it has been reported that combining exosomal miR-1262 and lncRNA RP11-513I15.6 with mRNA-RAB11A in the prediction of the development of HCC demonstrates high accuracy [201].

Fourth, other types of exosomal nucleic acid molecules. The exosomal circular RNA produced by HCC cells is involved in the progression and metastasis of HCC. For example, compared with healthy people, the expression of exosomal circPTGR1 in serum of HCC patients is significantly increased and positively correlated with tumor stage, indicating poor prognosis. Mechanism analysis shows that exosomal circPTGR1 produced by highly metastatic HCC cells can induce migration and enhance invasiveness of normal and low metastatic HCC cells, and circPTGR1 activates HCC cell migration and invasion via the miR-449a-MET signaling pathway [202]. The exosomal circ-deubiquitination (circDB) produced by adipocytes is significantly elevated in the serum of obese HCC patients. In vivo and in vitro experiments indicate that circDB can promote HCC cell proliferation and reduce DNA damage by downregulating miR-34 expression and inhibiting USP7 (ubiquitin-specific protease 7)/Cyclin A2 activation [15]. More and more data suggest that cell-free DNA (cfDNA) released into the bloodstream during the development of HCC and after treatment of HCC plays an important role as molecular marker for early diagnosis of HCC [203], efficacy evaluation [204], and prognosis judgment [205]. However, currently, research reports on HCC-related exosomal DNA are still rare and need to be further strengthened.

Exosomal proteins are also important markers in the diagnosis of HCC. Arbelaiz et al. analyzed the expression of exosomal proteins in the blood of HCC patients and healthy people, and found that G3BP and polymeric immunoglobulin receptor (PIGR) were significantly elevated in the exosomes of the former, and the prediction efficacy of these two proteins for HCC is superior to the widely used marker AFP [206]. Compared with healthy people, primary sclerosing cholangitis or cholangiocarcinoma patients, G3BP in exosomes of HCC patients is significantly higher. Therefore, G3BP can indicate the development of HCC earlier, and distinguish HCC from other liver-related diseases [20]. Hepcidin is an important regulator of iron metabolism in human, and iron overload is considered to be one of the important factors driving the development and progression of HCC [207]. Studies have found that the level of hepcidin mRNA in serum exosomes of HCC patients is significantly higher than that of healthy people, and then exosomal hepcidin may function as a diagnostic marker for HCC risk [208]. Clinical studies have also shown that elevated level of exosomal SMAD3 protein or mRNA in the serum of HCC patients can predict cancer progression and poor prognosis [147].

## Exosomes in preclinical studies for HCC treatment

Insensitivity or drug resistance to various chemotherapeutic drugs is a difficult problem in the treatment of HCC. Sorafenib is a first-line target drug for HCC. Although there are a large number of clinical trials confirming that the drug can effectively prolong the survival time of patients with advanced HCC, many patients showed sorafenib resistance, in which exosome-mediated apoptosis signal suppression and EMT are indicated to participate in drug resistance [209]. Zhen et al. found that exosomes produced by highly drug-resistant HCC activated hepatocyte growth factor (HGF)/c-MET/Akt pathway, and attenuated sorafenib-induced apoptosis [210]. Takahashi et al. found that drug treatment could induce increased expression of exosomal linc-RoR in HCC cells to inhibit p53 expression and reduce sorafenib-induced apoptosis [211]. Under the action of sorafenib, the expression of exosomal linc-VLDLR in HCC cells is increased, and linc-VLDLR is transported to adjacent cells via exosomes. After being taken up by target cells, linc-VLDLR can confer drug resistance to adjacent cells by upregulating ATP-binding cassette, sub-family G member 2 (ABC-G2) [212].

Tumor cells specifically pump anticancer drugs out of the cells, which is one of the causes of multi-drug resistance (MDR). Pgp-1 (P-glycoprotein-1/ABC-B1) expression is upregulated in HCC cells with MDR property, which confers tumor drug resistance [213, 214]. Both in vivo and in vitro experiments have shown that exosomes as drug carriers can bypass the Pgp-1-mediated drug efflux system and deliver drugs to the inside of tumor cells. This advantage is inextricably linked to the special uptake mechanisms of exosomes [215]. The efficacy of exoDOX, exosomes containing doxorubicin, is not different from that of doxorubicin treatment alone, while the cardiotoxicity caused by exoDOX is significantly lower than that of doxorubicin alone [216], indicating that exosomes as a drug carrier demonstrate certain targeting effect. Gap junction protein Cx43 (connexin 43) may be associated with this targeting effect [210]. Rivoltini et al. transfected K562 cells with lentivirus to introduce exogenous rhTRAIL into the cells. The exosomal rhTRAIL produced by these cells could effectively induce apoptosis in cells of various malignant tumors including HCC in vivo and in vitro, while showed no significant toxicity to normal cells [217]. Liang et al. introduced miR-26a into exosomes of renal cancer cells by electroporation. These exosomes could be taken up by HepG2 cells and downregulate Cyclin D2, Cyclin E2, and CDK6 level; induce HCC cell cycle arrest; and inhibit cell proliferation and metastasis [218].

The tumor suppressors contained in the exosomes have demonstrated great tumor inhibitory effects in in vivo and in vitro experiments. For example, exosomal miR-122 produced by HCC has the effects of inhibiting EMT, increasing drug sensitivity and inhibiting angiogenesis [219–221]. Adipose tissue-derived mesenchymal stem cells (AMSCs) that express miR-122 can induce G_0_/G_1_ arrest and apoptosis to enhance the chemosensitivity of HCC by transporting the exosomal miR-122 to HCC [222]. Moreover, when Huh7 cells showing upregulated miR-122 are co-cultured with HepG2 cells with low expression of miR-122, because miR-122 is transmitted between two cell lines by exosomes, the level of miR-122 in HepG2 is significantly increased, thereby effectively inhibiting the proliferation rate and invasiveness of HepG2 cells [223]. Studies have shown that IGF-1R may be a potential target of exosomal miR-122, which affects HCC chemosensitivity by regulating the expression of IGF-1R [224]. Wang et al. increased intratumoral miR-335-5p level by direct intratumoral injection of low doses of miR-335-5p exosomes that have tumor suppression effects, which could induce tumor growth arrest by down-regulating the expression of thrombospondin 1 and G-protein signaling 19 [13]. Zhang et al. injected exosomes containing miR-320a into rats via the tail vein, and then effectively inhibited HCC proliferation and metastasis by reducing the expression of pre-B-cell leukemia homeobox 3 in rats [225]. Therefore, intratumoral injection and i.v. injection are suitable exosome drug delivery pathways, providing a theoretical basis for future clinical applications.

Although exosomes can mediate the evasion of HCC cells from the immune system surveillance, exosomes also have good antigenicity and can activate immune responses. As an immunity inducing agent, the immune induction effect of exosomes is significantly superior to that of cell lysate [157]. For example, the abundant AFP in exosomes produced by in vitro cultured HCC can stimulate the antigen presentation function of dendritic cells (DCs), stimulate the proliferation of CD8^+^ T cells, regulate the secretion of inflammatory cytokines (reducing IL-10 and TGF-β secretion and increasing IFN-γ and IL-2 secretion), and enhance immune-induced apoptosis [157, 226]. Similar results have been obtained in in vivo experiments. For example, injection of AFP-containing exosomes derived from DC cells (DEXAFP) into primary HCC mice caused a strong specific immune response, induced aggregation of CD8^+^ T cells in tumors, and reduced tumor growth rate [227]. The injection of exosomes produced by adipose-derived mesenchymal stem cells into the HCC mice via the rat tail vein stimulated the inhibitory effects of NK cells on tumor growth [228]. The liver is an immune-tolerant organ, which poses certain difficulties for immunotherapy of HCC, while exosomes can bypass the immunosuppressive environment of the liver, and then demonstrate strong advantages [229, 230]. Under the action of chemotherapeutic drugs, HCC cells can release exosomes containing HSPs (such as HSP60, 70, and 90), and activate the cytotoxicity of human NK cells to induce anticancer effects. Even HCC-resistant drugs (such as carboplatin and irinotecan hydrochlorid) can still activate HCC cells to produce HSPs containing exosomes, which can upregulate the expression of CD69, NKG2D, NKp44 and other proteins in NK cells, but downregulate inhibitory receptor CD94 expression in NK cells, increase granzyme B production, and activate the NK cell cytotoxic response [22]. Therefore, this type of drug induced exosomes can be used as a potential HCC treatment vaccine.

According to the literature, no studies of clinical trials assessing the use of exosomes for the treatment of liver cancer have been performed. Some clinical studies of exosome treatment for cancers other than HCC were carried out around 2000. For example, in a phase I clinical trial, Escudier et al. injected exosomes derived from autologous DCs into patients with metastatic melanoma intradermally and subcutaneously. Although the efficacy was not satisfactory, no patient developed obvious toxic or side effects, indicating that exosomes have a certain safety and feasibility for the treatment of metastatic melanoma [231]. In another phase I clinical trial, Morse et al. successfully activated immune responses in non-small cell lung cancer (NSCLC) patients using exosomes produced by DCs, which delayed the progression of NSCLC to some extent, and two of the patients even saw cessation of tumor progression for more than 12 months [232]. In a phase II clinical trial for patients with NSCLC, patients whose chemotherapy had failed were injected with exosomes derived from DCs. These NK cells were activated in vivo and the treatment achieved good efficacy [233]. In a phase I clinical trial performed on colon cancer patients, Dai et al. found that exosomes existing in malignant ascites together alongside granulocyte-macrophage colony-stimulating factor (GM-CSF) activated cytotoxic T lymphocytes in patients and induced tumor-specific immune killing effect [234]. Two other phase I clinical trials are ongoing (please refer to http://www.clinicaltrials.gov for colon cancer: NCT01294072; head and neck cancer: NCT01668849).

The use of exosomes as a carrier in the clinical cancer treatment including liver cancer still has many difficulties that must be overcome. First, the homogeneity of exosomes is difficult to be guaranteed. Exosomes are a group of extracellular vesicle mixture produced by cells. Only a few types of them have therapeutic tumor inhibitory effects, while the molecular substances contained in other types of exosomes may not have therapeutic effects or may even promote tumor progression. Second, the exosomes are mainly administrated by subcutaneous injection. Although this method is simple and easy to perform, the absorption efficiency is not satisfactory. Third, the exosome extraction methods are limited and of low efficiency, so it is difficult to prepare exosomes in large quantities. Especially for exosome-mediated tumor immunotherapy, it is extremely important to ensure that the quantity of exosomes used is sufficient to elicit an effective tumor immune response. With the improvement of identification, isolation and purification techniques, we believe exosomes will be widely used in the clinical treatment of liver cancer.

## Conclusions and future perspectives

Exosomes are microvesicles necessary for the human body to maintain its own stability. Proteins, nucleic acids, and other substances contained in exosomes are present in a relatively independent special environment, having a high stability and abundance than tumor markers freely existed in tissues or body fluids, and therefore, possessing unique advantages and important clinical application potential in the early diagnosis and treatment of HCC. Isolation, purification, component identification, and function analysis of HCC-related exosomes will benefit the follows: first, it will help to clarify the mechanism of HCC development and progression, and identify specific signal targets for intervention therapy; second, microcapsules with tumor suppressor function of similar structures can be synthesized in vitro, and re-introduced into the body for the purpose of treatment; third, anticancer drugs can be encapsulated into the exosomal vesicles, and directly kill the cancer cells by intravenous injection or intratumor injection into the body, avoiding the normal cell or organ toxicity caused by the free diffusion of drugs; forth, specific antigens can be labeled to the outer membrane of the exosomes to induce a specific immune response, achieving anti-tumor effects; fifth, exosomes can be used as a novel tumor marker to play a role in the non-invasive (extreme) early diagnosis of HCC based on liquid biopsy, among which, non-coding RNA is stable in nature, and is more sensitive and specific compared with traditional protein markers such as AFP. Furthermore, the accurate efficacy assessment and prognosis of advanced HCC treatment cannot be achieved by currently commonly used imaging examinations. Surgical treatment, radiotherapy, and chemotherapy will stimulate the body or tumor tissue to produce specific exosomes. Therefore, apoptosis-related exosomes, necrotic-related exosomes, or angiogenesis-related exosomes can be used as reliable prediction markers for HCC efficacy and prognosis assessment, and deserve further development into reagent kit. However, there are still many problems regarding identification, isolation, and purification of exosomes, unclear molecular mechanisms, and immature artificial synthetic exosome techniques. Many studies on the diagnosis and treatment of HCC are still in the pre-clinical experimental stage, and there is still a long way to go for practical applications.

## Data Availability

All the materials and data supporting the conclusions of this review are included within the article.

## Abbreviations

ABC-G2: ATP-binding cassette, sub-family G member 2
AFP: Alpha fetoprotein
ALD: Alcoholic liver disease
B4GALT3: β-1,4-galactosyltransferases III
CAF: Cancer-associated fibroblast
CeRNA: Competing endogenous RNAs
CHB: Chronic hepatitis B
CSC: Cancer stem cell
CTC: Circulating tumor cell
DC: Dendritic cell
ECM: Extracellular matrix
EMT: Epithelial-mesenchymal transition
ESCRT: Endosomal-sorting complex required for transport
EVs: Extracellular vesicles
HBV: Hepatitis B virus
HCC: Hepatocellular carcinoma
HCV: Hepatitis C virus
HGF: Hepatocyte growth factor
HIF-1α: Hypoxia inducible factor-1α
HRS: Hepatocyte growth factor regulated tyrosine kinase substrate
HSC: Hepatic satellite cell
HUVEC: Human umbilical vein endothelial cell
IGFR: Insulin-like growth factor receptor
ILVs: Intraluminal vesicles
LC: Liver cirrhosis
LncRNA: Long non-coding RNA
MVB: Multivesicular body
NAFLD: Non-alcoholic fatty liver disease
NASH: Non-alcoholic steatohepatitis
NKC: Natural killer cell
NSMase: Neutral sphingomyelinase
PDCD4: Programmed cell death protein 4
PRR: Pattern recognition receptor
Rac1: Ras-related C3 botulinum toxin substrate 1
SMPD2/3: Sphingomyelin phosphodiesterase 2/3
STAM: Signal-transducing adaptor molecule
TAM: Tumor-associated macrophage
TGF-β: Transforming growth factor-β
TLR3: Toll-like receptor 3
ZEB: Zinc finger E-box binding homeobox
